# Supplementary material for: The influence of kindness on academics’ identity, well-being and stress
Source: PLoS One. 2024 Oct 22;19(10):e0312269. doi: 10.1371/journal.pone.0312269 (PMC11495609; doi:10.1371/journal.pone.0312269)
Supplement: S1 Table — (PDF) [file pone.0312269.s001.pdf]

Supplemental Table 1. Percentage, frequency, and examples of dignity affirmations for all 10 categories identified in the written responses

	%	N (N = 182)
The agent of kindness acted in ways that made receiver feel safe (as opposed to being threatening)<br><br>Example: "During my PhD program, I failed my qualifying exam. I thought of dropping out but had a meaningful conversation with a mentor. There was so much about the culture of science I wanted to change, and I felt like it wasn't for me. He used the metaphor of learning ballet before doing contemporary (I was into dance in my spare time), and that really resonated with me. I felt motivated to learn about the system I wanted to change. What I appreciated most about this interaction, though, was his willingness to listen to me and ask me questions to help me figure out for myself whether or not I wanted to finish my degree. I got the sense he really wanted to understand my perspective and would respect whatever decision I made for myself."	54.4	99
The agent of kindness acknowledged the validity of the receiver's feelings, concerns/experiences<br><br>Example: "As someone with disabilities, I had a very validating moment with one of my professors (for my proofs class) who shared a personal anecdote saying that she really hopes I do not feel incompetent for being a minority by not only ethnicity, but also disability. Given the lack of representation of STEM students with disabilities, this felt especially meaningful because it was during my first semester at a new university."	53.9	98
The agent of kindness positively recognized the receiver's efforts, thoughtfulness and/or talents<br><br>Example: "A professor that I really looked up to talked really highly about me to a conservation group and got me a meeting with them and the leaders of that agency to discuss about my ideas and future collaborations. It felt really validating and decreased my imposters syndrome to feel that someone as intelligent and well respected as this professor would think so highly of my ideas."	50.6	92
The agent of kindness conveyed inclusion to the receiver<br><br>Example: "A new colleague spent much time making sure I understood the culture of the school I was starting at. I appreciated her time and insight. I see it as a way of lifting others up, if they succeed, we all succeed."	36.3	66
The receiver described the act of kindness as sincere<br><br>Example: "I had a math professor at Wellesley who reached out and helped me apply to REUs. She did not have to do this. She helped me identify opportunity and wrote me	34.6	63

a support letter for the programs. I got into one at Mount Holyoke College that was in applied Math studying fluid dynamics. That REU was instrumental to helping me realize I didn't want to do pure math, but rather physics. It was an essential step on my career.”		
The agent of kindness treated the receiver fairly<br><br>Example: “I had just started a doc program and was 42. I felt out of place as an older student, a mother, a woman of color, and hadn,Äôt been a student in 17 years. But I was determined and curious. I asked a lot of question as I tried to understand concepts, academic processes and culture. A faculty member who became my adviser and later my chair, was patient and very transparent in answering my questions. Never made me feel like I was stupid or like my questions were stupid. That felt like the most kind thing anyone could do for me.”	28.0	51
Agent of kindness made an effort to understand the receiver's point of view<br><br>Example: “As a first-year PhD student, I was nervous to tell my advisor I might need to leave with no notice since my grandma was sick. He accepted it with no question and told me "This is just your job. Family and health come first." It was reassuring to hear that early in my career and know he would support me.”	26.4	48
The agent of kindness made gave the receiver the benefit of the doubt<br><br>Example: “A professor forgave me for lying in a spur-of-the-moment decision when I was in a hurry. He said in an email that he understood the error and I was grateful that he was willing to forgive.”	14.9	27
The agent of kindness gave the receiver the freedom to express their authentic self<br><br>Example: “When I was in my second year of grad school, I was dealing with a difficult situation with my advisor in which I was asked to leave the group and I felt I was the victim of sexism. I sought advice from a postdoc who had recently defended his PhD thesis in my research group, and he spent a lot of time processing my experience with me. It felt very validating when he told me that what happened to me was not fair, and he spent time strategizing with me about how to navigate the situation moving forward. It was memorable to me because if he hadn't put the time and effort into investing in my success at that moment, I likely would have left the physics department.”	8.2	15
The agent of kindness respected receiver's freedom of choice<br><br>Example: “As a first-year PhD student, I was nervous to tell my advisor I might need to leave with no notice since my grandma was sick. He accepted it with no question and told me "This is just your job. Family and health come first." It was reassuring to hear that early in my career and know he would support me.”	6.0	11
The agent of kindness apologized when they violated the receiver's dignity	0	0
