# Supplementary material for: The influence of kindness on academics’ identity, well-being and stress
Source: PLoS One. 2024 Oct 22;19(10):e0312269. doi: 10.1371/journal.pone.0312269 (PMC11495609; doi:10.1371/journal.pone.0312269)
Supplement: S3 File — (PDF) [file pone.0312269.s004.pdf]

Kindness Scales

WHAT YOU GIVE TO OTHERS [Kindness Given Scale]

When there was opportunity in the past month, how often did you engage in these actions?
[1 - Never, 2 - Almost Never, 3 - Sometimes, 4 - Almost every time, 5 - every time]

1. Gave others the freedom to express their authentic selves without fear of you negatively judging them
2. Positively recognized others' efforts, thoughtfulness and/or talents
3. Acknowledged the validity of others' feelings, concerns and experiences
4. Conveyed inclusion (e.g., in your family, team, club, community, profession, etc.)?
5. Acted in ways that made others feel safe around you (as opposed to being threatening)
6. Treated others fairly
7. Respected others' freedom of choice
8. Made an effort to understand others' point of view
9. Gave others the benefit of the doubt
10. Apologized when you violated others' dignity in some way
11. Treated others with kindness *[used as a manipulation check, not included in the scale]*

WHAT YOU RECEIVE FROM OTHERS [Kindness Received Scale]

When there was opportunity in the past month, how often did you have these experiences?
[1 - Never, 2 - Almost Never, 3 - Sometimes, 4 - Almost every time, 5 - every time]

1. Felt free to express your authentic self without being negatively judged
2. Your efforts, thoughtfulness and/or talents were positively recognized
3. Your feelings, concerns and experiences were acknowledged as valid
4. Others conveyed you were included (e.g., in family, team, club, community, profession, etc.)
5. Others actions made you feel safe with them
6. You were treated fairly
7. Your choices were respected
8. Others made an effort to understand you
9. You were given the benefit of the doubt
10. You received an apology when your dignity felt violated
11. You experienced kindness from others *[used as a manipulation check, not included in the scale]*
