# Supplementary material for: The influence of kindness on academics’ identity, well-being and stress
Source: PLoS One. 2024 Oct 22;19(10):e0312269. doi: 10.1371/journal.pone.0312269 (PMC11495609; doi:10.1371/journal.pone.0312269)
Supplement: Attachment — Submitted filename: Response to Reviewers.pdf [file pone.0312269.s005.pdf]

UNIVERSITY of HAWAIʻI®
MĀNOA

John A. Burns School of Medicine
Department of Native Hawaiian Health
Native Hawaiian Center of Excellence

September 4th, 2024

Dear PLOS ONE Editor and Reviewers,

We would like to thank you for the thorough and constructive feedback provided on our manuscript titled "The influence of kindness on academics' identity, well-being, and stress" (Manuscript ID: PONE-D-24-15380). We have carefully considered each of the comments and have revised the manuscript to address the concerns raised. We believe that these revisions have significantly improved the quality of our work, and we are grateful for the opportunity to make these improvements.

Please find below our detailed responses to the comments, organized by reviewer. The revised manuscript, with all changes highlighted, is also attached.

Detailed response to comments

Editor:

1. **Comment:** Please ensure manuscript meets PLOS ONE style requirements.
    a. **Response:** We have carefully reviewed and applied PLOS ONE's style templates.
    b. **Action Taken:** All headers, tables, figures, and supplemental materials have been revised to meet the requirements.

2. **Comment**: Funding should not appear in the acknowledgements section
    a. **Response**: The funding information was in both the acknowledgements section and the funding statement.
    b. **Action Taken:** Funding information was removed from the acknowledgements section.

3. **Comment**: Please include your full ethics statement in the methods section of the manuscript, including the full name of the IRB committee.
    a. **Response**: The requested information is now included in the Methods section
    b. **Action Taken:** The following was included in the Methods section: "The study was reviewed and approved by the University of California San Francisco Mount Zion Committee Institutional Review Board (#21-35884). Written informed consent was obtained from all study participants."

4. **Comment:** Please include captions for your supporting files at the end of your manuscript.
    a. **Response:** Added a caption at the end of the manuscript file.
    b. **Action Taken:** The following was added at the end of the manuscript in the Supporting Information section: "S1 Table. Percentage, frequency, and examples of dignity affirmations for all 10 categories identified in the written responses."

5. **Comment:** Please review your reference list to ensure that it is complete and correct.
    a. **Response:** All references were checked for completeness.

    b. **Action Taken:** Reference 23 was updated with final publication information: Estrada M, Hosoda KK. Leading with Heroic Kindness. Encyclopedia of Heroism Studies. Springer Cham; 2023. Available: https://doi.org/10.1007/978-3-031-17125-3

6. **Additional Change**
    a. The author, K. Kanoho Hosoda, has started a new position at the University of Hawaiʻi at Mānoa. The author affiliation and contact information has been changed accordingly.

Reviewer 1:

1. **Comment:** There is a lack of connection between line 51 and 52
    a. **Response:** The author agrees with the comment.
    b. **Action Taken:** The second clause was removed from the sentence in line 51. The two sentences now read: "A variety of definitions of an act of kindness exists in the literature [18,19]. The emphasis in these definitions is on the nature of the action done."
2. **Comment:** The theoretical framework does not address the power hierarchy that may exist between those working in academia versus those who are studying in academia.
    a. **Response:** The author agrees there is a power hierarchy that is not addressed.
    b. **Action Taken:** The power hierarchy is address in the discussion section, see comment below.
3. **Comment:** It is necessary to add a figure or table with means and standard deviations of the variables under study
    a. **Response:** The author agrees that the addition of a table with means and standard deviations would improve the manuscript.
    b. **Action Take:** Table 1. Summary of sample descriptive statistics was included.
4. **Comment:** If potential differences between workers and students are not going to be addressed, it could be included as a future line of research. This may make more sense than the topic of aggressions.
    a. **Response:** The author agrees that the differences between workers and students should be addressed in future work.
    b. **Action Taken:** The following was included in the discussion section of the manuscript:
        "We acknowledge that increased variability may exist in this sample of academics, which includes both higher education students and people who work in higher education from various fields of study. We recognize there is a power hierarchy between students, staff, and faculty members in higher education that may influence outcomes. While this study did not directly address the power hierarchy within higher education, future studies would benefit from comparing these different communities within higher education environments."

Reviewer 2:

1. **Comment(s):** Formatting discrepancies including: 1) consistency of the hyphen in well-being, 2) font size, 3) italicizing research question, 4) extra spacing
    a. **Response:** Author acknowledges the errors in formatting
    b. **Action Taken:** Paper was reformatted using PLOS ONE style requirements and copy edited for hyphen consistency, font size, italics, and spacing.
2. **Comment:** The study stated that 21.4% were in STEM fields but did not include 78.6%. Who was part of 78.6%? The total needs to add up to 100%.
    a. **Response:** Percentages did not add up due to rounding errors
    b. **Action Taken:** All percentages and numbers were rounded to the nearest hundredth.

3. **Comment:** Ethnic Demographic Distribution - the total does not add up to 100% (lines 177-180)
    a. **Response:** Percentages did not add up due to rounding errors
    b. **Action Taken:** All percentages and numbers were rounded to the nearest hundredth.
4. **Comment:** Table 2 (line 309): Highlight the rows and columns interchangeability so that they are readable, and remove the border at the last row, "Note:..."
    a. **Response:** We recognize the error and acknowledge highlighting will improve readability
    b. **Action Taken:** Rows were highlighted interchangeably for the table, which is now Table 3.
5. **Comment:** Line 386 - need to confirm whether it should be N=162 or N=182 (Overall, (N=162),...)
    a. **Response:** We agree it the sample was unclear.
    b. **Action Taken:** We included the percentage and further explanation as follows: “Notably, 89.01% of the participants (N=162 of the 182 study participants) listed words...”
6. **Comment:** The total does not add up to 100% (line 403)
    a. **Response:** The author does not agree with the comment, as the total adds up to 100.
    b. **Action Taken:** None. 41.98+22.22+12.96+11.73+11.11= 100
7. **Comment:** Figure 1 diagram - Paths labeled Dgx & Drx in the description should be different, not the same.
    a. **Response:** We agree with the comment
    b. **Action Taken:** Path labels were revised in the figure.
8. **Comment:** Figure 2 diagram - the word Kindness Given should be labeled instead of Kindness Action
    a. **Response:** We agree with the comment.
    b. **Action Taken:** The label Kindness Action was revised to Kindness Given.
9. **Comment:** Supplemental Table 1 - font type needs to be consistent throughout
    a. **Response:** We agree the supplemental table needed to be revised.
    b. **Action Taken:** Formatting including font type and size were revised. Additionally, all percentages were rounded to the nearest hundredth instead of the nearest tenth to maintain consistency throughout the paper.

We express our sincere gratitude to the reviewers and the editor for your thorough and insightful comments on our manuscript. We appreciate the opportunity to improve the manuscript based on your feedback.

We hope that the revised manuscript now meets the standards of PLOS ONE and addresses all the concerns raised. However, if there are any additional questions or further revisions needed, we are more than willing to address them promptly.

Thank you again for your time and consideration. We look forward to your favorable response.

Respectfully,

K. Kanoho Hosoda

K. Kanoho Hosoda, Ph.D.

Assistant Professor
Department of Native Hawaiian Health
John A. Burns School of Medicine
University of Hawaiʻi at Mānoa
651 Ilalo Street Honolulu, HI 96813
